# Stationary-Phase Mutagenesis in Stressed *Bacillus subtilis* Cells Operates by Mfd-Dependent Mutagenic Pathways

**DOI:** 10.3390/genes7070033

**Published:** 2016-07-05

**Authors:** Martha Gómez-Marroquín, Holly A. Martin, Amber Pepper, Mary E. Girard, Amanda A. Kidman, Carmen Vallin, Ronald E. Yasbin, Mario Pedraza-Reyes, Eduardo A. Robleto

**Affiliations:** 1 Department of Biology, Division of Natural and Exact Sciences, University of Guanajuato, Noria Alta S/N, Noria Alta, Guanajuato 36050, México; qfb.mgomez@gmail.com; 2 School of Life Sciences, University of Nevada, 4505 Maryland Parkway, Las Vegas, NV 89154, USA; microbiologymartin@gmail.com (H.A.M.); Amber.m.pepper@gmail.com (A.P.); girard@bcm.edu (M.E.G.); prisbre8@unlv.nevada.edu (A.A.K.); vallincarmen@gmail.com (C.V.); 3 College of Arts and Sciences, University of Missouri-St Louis, 303 Lucas Hall, One University Boulevard, St. Louis, MO 63121, USA; yasbinr@umsl.edu

**Keywords:** transcription-mediated mutagenesis, Mfd, NER, MutY, PolI

## Abstract

In replication-limited cells of *Bacillus subtilis*, Mfd is mutagenic at highly transcribed regions, even in the absence of bulky DNA lesions. However, the mechanism leading to increased mutagenesis through Mfd remains currently unknown. Here, we report that Mfd may promote mutagenesis in nutritionally stressed *B. subtilis* cells by coordinating error-prone repair events mediated by UvrA, MutY and PolI. Using a point-mutated gene conferring leucine auxotrophy as a genetic marker, it was found that the absence of UvrA reduced the Leu^+^ revertants and that a second mutation in *mfd* reduced mutagenesis further. Moreover, the *mfd* and *polA* mutants presented low but similar reversion frequencies compared to the parental strain. These results suggest that Mfd promotes mutagenic events that required the participation of NER pathway and *PolI*. Remarkably, this Mfd-dependent mutagenic pathway was found to be epistatic onto MutY; however, whereas the MutY-dependent Leu^+^ reversions required Mfd, a direct interaction between these proteins was not apparent. In summary, our results support the concept that Mfd promotes mutagenesis in starved *B. subtilis* cells by coordinating both known and previously unknown Mfd-associated repair pathways. These mutagenic processes bias the production of genetic diversity towards highly transcribed regions in the genome.

## 1. Introduction

Stationary-phase mutagenesis (SPM), also referred to as stress-induced mutagenesis, is the collection of cellular processes that produces genetic alterations in non-growing cells. These processes are well conserved, have been implicated in genome instability, and are viewed as mechanisms that produce genetic diversity and speed evolution in times of stress [1,2,3,4]. It is possible that during conditions of stress or in nutrient-limited cells mechanisms that bias mutagenic events towards highly transcribed genes are activated, thus increasing the adaptive potential of mutations [5,6,7].

In 1966, Witkin and coworkers described for the first time a genetic factor that prevented the formation of mutations induced by exposure to ultraviolet light (UV). *Escherichia coli* cells proficient in this factor expressed a mutation frequency decline (*mfd*) phenotype [8]. Subsequent studies showed that Mfd is a key component of transcription-coupled repair (TCR), which directs the nucleotide excision repair (NER) system to UV-lesions occupying the template strand in transcribed regions [9]. In *E. coli*, genetics and biochemical evidence indicates that Mfd mediates high-fidelity repair of DNA lesions or removal of obstacles that block the progress of the RNA polymerase (RNAP) during active transcription [10,11,12,13,14]. At stalled or blocked RNAP-DNA complexes, Mfd first binds RNAP, then dissociates the transcription elongation complex and finally recruits proteins of the NER system. Recruitment of UvrAB_2_ complex initiates incision of the template strand and enlists UvrC to the site of damage. The UvrBC complex generates incisions flanking the transcription-stalling DNA lesion. UvrD, a helicase, removes the ~13 b DNA fragment containing the lesion and generates a single-stranded gap to be filled by DNA PolI and sealed by ligase A [15]. Currently, structural and single-molecule resolution experiments are investigating how Mfd interacts with RNAP, whether it translocates to the lesion, and how it recruits Uvr proteins [13,14].

It has been also proposed that Mfd may recruit DNA repair proteins, other than the UvrAB_2_ complex from the NER system, to the template strand [14]. In non-replicating *E. coli* cells, Mfd prevented transcriptional bypass of DNA templates containing lesions of oxidative nature or DNA-repair intermediates generated by proteins that eliminate ROS-promoted DNA damage (known to be part of the base excision repair system (BER)) [16,17], which raises the possibility that Mfd may interact with components of the BER system, specifically with DNA glycosylases or apurinic/apyrimidinic (AP)—endonucleases that recognize these types of lesions [18,19].

In the context of mutagenesis, previous experiments showed that Mfd deficiency had no effect on the reversion frequency of a *tyr* allele in *E. coli* cells starved for tyrosine [20]. It has been shown that Mfd promotes origin-independent replication restarts and formation of recombination intermediates, and that these events may generate point mutations or amplifications [7]. In *Bacillus subtilis*, a recent report showed that Mfd, the NER system and PolY1, an error-prone DNA polymerase, are all part of a mutagenic pathway that prevents conflicts between transcription and replication [21].

In starved or replication-limited *Bacillus subtilis* cells, Mfd is mutagenic. However, the molecular basis of such a process remains unknown [22]. It is well established that stationary-phase *B. subtilis* cells halt DNA replication [23,24,25]. Then, one would expect that the mutagenic contribution of conflicts between transcription and replication is severely reduced or abrogated. In addition, while Mfd is required for stationary-phase mutagenesis [22], recombination functions are not [26]. This is in stark contrast to how stress-induced mutagenesis occurs in *E. coli*, in which recombination-repair functions are necessary to produce mutations (reviewed in [2,27]).

An idea considered in the present study is that Mfd generates stationary-phase mutations through its TCR activities and error-prone DNA synthesis. Recent reports have associated UvrD with a TCR process; in this mechanism, the helicase activity of UvrD operates to backtrack stalled RNAP at DNA lesions, this event then uncovers DNA damage to be accessed by repair systems [28]. Moreover, NusA, proposed to take part in UvrD-mediated TCR [28], and error-prone DNA synthesis, have been associated with stress-induced mutagenesis in *E. coli* [29,30].

Another possibility addressed here was the association of Mfd with BER proteins, specifically the DNA glycosylase MutY, which has been implicated in the generation of stationary-phase mutations in *B. subtilis* [31]. In this regard, it has been reported that accumulation of base mismatches saturates the mismatch repair (MMR) capacity and promotes stationary-phase mutagenesis through a mechanism that involves processing of A-C or A-G mismatches by MutY [31].

Here we present evidence indicating that Mfd combines with UvrA, MutY and PolI in the formation of mutations at *leuC427* mutant allele and therefore expanding the transcription-mediated mutagenic functions of Mfd beyond NER in replication-limited cells.

## 2. Materials and Methods

### 2.1. Bacterial Strains and Growth Conditions

Strain YB955 is a prophage-“cured” *B. subtilis* strain 168 containing point mutations in three genes, *metB5* (ochre), *hisC952* (amber), and *leuC427* (missense) [26]. *B. subtilis* strains employed in this study (Table 1) were routinely isolated on tryptic blood agar base (TBAB) (Acumedia Manufacturers, Inc., Lansing, MI, USA), and liquid cultures were grown in Penassay broth (PAB) (antibiotic medium 3, Difco Laboratories, Sparks, MD, USA) supplemented with 1X Ho-Le trace elements [32]. *E. coli* cultures were grown in Luria-Bertani (LB) medium. When required, tetracycline (Tet; 10 μg·mL^−1^), spectinomycin (Sp; 100 μg·mL^−1^), ampicillin (Amp; 100 µg·mL^−1^), chloramphenicol (Cm; 5 μg·mL^−1^), erythromycin (Em; 1 μg·mL^−1^) or isopropyl-β-D-thiogalactopyranoside (IPTG; 1 mM) was added to media.

### 2.2. Construction of Mutant Strains

*B. subtilis* strains carrying single, double and triple mutations were generated as indicated in Table 1, employing standard techniques.

The pBT and pTRG vectors containing *mutY* (PERM1084) and *mfd* (PERM1072) genes were generated as mentioned below. The *mutY* and *mfd* ORFs were first amplified by PCR using chromosomal DNA purified from *B. subtilis* YB955 with the following set of oligonucleotide primers: for *mutY*, 5′-CGGGATCCATGAACGTACTTGAAGAC-3′ (forward) and 5′-CGCTCGAGCGGAGCAGCCGAGATGGC-3′ (reverse); for *mfd*, 5′-CGGGATCCATGGACAACATTCAAACC-3′ (forward) and 5′-CGCTCGAGCGTTGATGAAATGGTTTG-3′ (reverse). Primers were designed to insert *BamH*I/*XhoI*I sites in both cases (sequences underlined). Amplifications were performed with Vent DNA polymerase (New England BioLabs, Beverly, MA, USA). PCR products purified from a low-melting-point agarose gel were ligated into PCR-BluntII-TOPO (Invitrogen, Carlsbad, CA, USA). The resulting constructs were treated with *Not*I/*Xho*I and *Bam*HI/*Xho*I to release *mutY* and *mfd* ORFs. The purified fragments were ligated into pBT and pTRG to generate the constructs PERM1084 and PERM1072. The correct orientation of the inserted genes was verified by restriction analysis.

### 2.3. Stationary-Phase Mutagenesis Assays

The stationary-phase mutagenesis assay was conducted as described by Sung et al. [26]. Cultures were grown in flasks containing antibiotic PAB and Ho-Le trace elements [32] with aeration (250 rpm) at 37 °C until 90 min after the cessation of exponential growth (designated T_90_ (90 min after the time point in the culture when the slopes of the logarithmic and stationary phases of growth intercepted). Growth was monitored with a spectrophotometer measuring the optical density at 600 nm (OD_600_). Cells were harvested by centrifugation (10,000 × *g*, 10 min), resuspended in 10 mL of Spizizen minimal salts (SMS) [36] and plated in quintuplicate onto solid Spizizen minimal medium (SMM; 1× Spizizen salts supplemented with 0.5% glucose, 50 µg·mL^−1^ of each methionine, histidine, isoleucine and glutamic acid, and 200 ng·mL^−1^ of leucine). To induce *mutY* and *mfd* expression, the selection medium was supplement with 1 mM IPTG mL^−1^. The plates were incubated for 9 days. Each day plates were scored for the appearance of Leu^+^ colonies. The initial titer of *B. subtilis* cells was determined by serially diluting the resuspended culture and spread plating on SMM containing 50 mg/mL of histidine, methionine, leucine, isoleucine, and glutamic acid. Colonies were counted after 48 h of incubation. The initial titers were used to normalize the cumulative number of revertants per day to the number of CFU plated. Assays were repeated at least three times for each tested strain.

### 2.4. Analysis of Mutation Rates

The growth-dependent reversion rates for Leu were measured by fluctuation tests with the Lea-Coulson formula, r/m-ln(m) = 1.24 [37]. Three parallel cultures were used to determine the total number of CFU plated on each plate (Nt) by titration. The mutation rates were calculated as previously described with the formula m/2Nt [26,38,39].

### 2.5. Beta-Galactosidase Assays

*B. subtilis* strains PERM1123, containing a transcriptional *mfd-lacZ* fusion (Table 1), were propagated in liquid PAB medium. Aliquots of 1.5 mL were collected from cultures at exponential growth phase and stationary phase and processed for determination of β-galactosidase activity as previously described [40].

### 2.6. Quantitative Real-Time PCR

Total RNA was isolated from exponentially growing (0.5 of OD_600nm_) and stationary-phase (T_90_) *B. subtilis* YB955 cells grown in A3 medium was isolated by using TRIzol reagent (TRI reagent, Molecular Center, Inc., Cincinnati, OH, USA). The mRNA isolated was reverse transcribed and amplified for real-time PCR using a One-Step SYBR Green qRT-PCR (Quanta, Biosciences, Beverly, MA, USA). Master mixes of 25 µL of One-Step SYBR Green qRT-PCR containing 50 ng of RNA and 300 nM final concentration of *mfd* primers (forward, 5′-AGAAGAATGCTGCCGCCTGTT-3′ and reverse 5′-CCGCTCATACCCTACCTCTACCAG-3′, 117-bp amplicon) or *veg* primers (forward, 5′-TGGCGAAGACGTTGTCCGATA-3′ and reverse 5′-CGGCCGCCGTTTGCTTTTAAC-3′, 82-bp amplicon). Three replicates from each culture condition were assayed and normalized to the expression of the internal control gene *veg* [41,42]. One reaction was assessed with no reverse transcriptase and no-template as controls. Quantitative real-time PCR was run on a Bio-Rad iCycler iQ Real-Time PCR Detection System (Bio-Rad, Hercules, CA, USA), using the manufacturer’s suggested protocol and an annealing temperature of 60 °C. Results were calculated by the 2^−ΔΔCT^ (where C_T_ is threshold cycle) method for relative fold expression [43].

## 3. Results

### 3.1. Transcription-Coupled Nucleotide Excision Repair Produces Stationary-Phase Mutations at leuC427 gene

**Figure 1:**
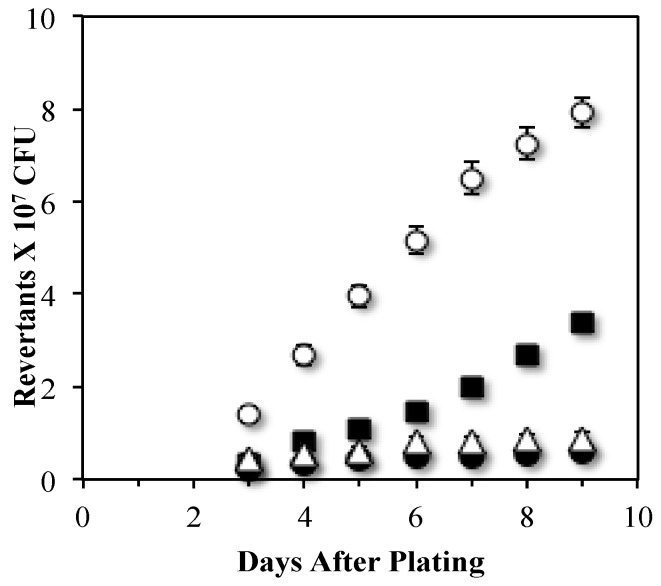
Mfd works with UvrA to produce stationary phase mutations. Stationary-phase-induced reversions to *leu* mutant allele of the YB955 (parental strain) (○), PERM687 (*uvrA*) (■), YB9801 (*mfd*) (●) and HAM300 (*uvrA mfd*) (Δ) *B. subtilis* strains were determined as described in Material and Methods. Data represent the average of three separate tests ± standard error of the mean (SEM).

We constructed mutants lacking Mfd and UvrA and assayed the strains’ ability to produce stationary-phase leucine revertants (Leu^+^). Results revealed that whereas disruption of *mfd* completely abolished the production of Leu^+^ revertants, the absence of UvrA only promoted a partial decrease in this mutagenic event (Figure 1). Of note, the reversion frequency values to *leuC* were similar in the *mfd* and *mfd uvrA* mutants (Figure 1), suggesting that Mfd was epistatic onto UvrA to produce stationary-phase Leu^+^ revertants. Therefore, transcription-coupled nucleotide excision repair is only partially involved in SPM and pointed to the role of additional factors interacting with Mfd to generate mutations in conditions of nutritional stress.

**Figure 2:**
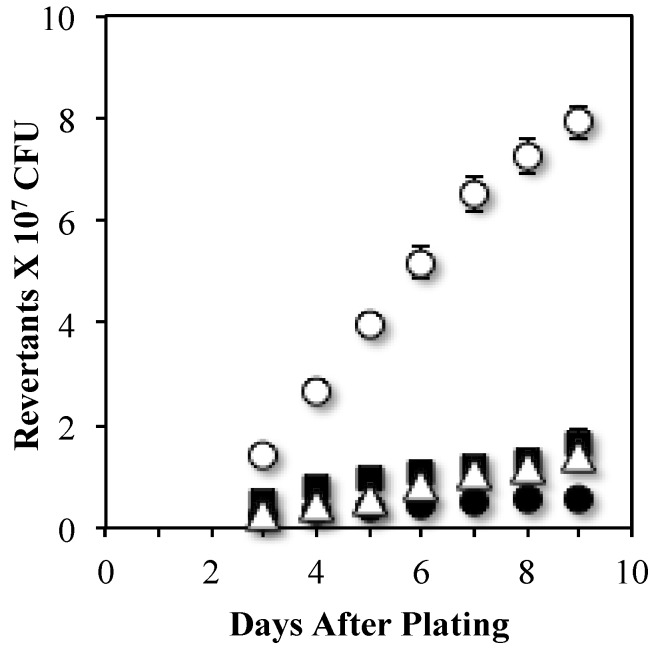
The stationary-phase mutagenesis requires PolI. The accumulation of Leu^+^ revertants of the YB955 (parental strain) (○), AMP100 (*polA*) (■), YB9801 (*mfd*) (●), and AMP101 (*polA mfd*) (Δ) *B. subtilis* strains were obtained as described in Materials and Methods. Data represent the average of three separate tests ± standard error of the mean (SEM).

Because PolI executes the DNA synthesis step during transcription-coupled repair, we inquired whether this polymerase is involved in Mfd-mediated Leu^+^ SPM. Strains lacking Mfd or PolI were measured for their ability to accumulate Leu^+^ revertants. Results showed that in reference to the parental strain YB955, the *polA* mutant presented very low but similar levels of Leu^+^ revertants as those produced by the Mfd-deficient strain (Figure 2). These results suggest that Mfd and PolI are part of a pathway additional to and different than TCR that generates mutations at *leuC427* in stationary phase cells. Of note, the levels of SPM-dependent Leu^+^ reversions shown in Figure 1 and Figure 2 cannot be attributed to viability levels of the strains (Figure S1).

### 3.2. An Mfd Mutagenic Pathway Operates through MutY Activity Producing Leu^+^ SPM

As noted above, the NER pathway (UvrA) partially contributed in generating stationary-phase Leu^+^ reversions suggesting that Mfd-mediated SPM proceeds through both transcription-coupled nucleotide excision repair and an alternate, yet-to-be known mechanism(s). Previous results revealed that the mutagenic changes occurring at *leuC427* in the strain YB955 during stationary phase are not generated via oxidative damage to DNA bases [31,44]. However, a subsequent set of experiments showed that such reversion events depended on the activity of the MutY DNA glycosylase, a component of the oxidized guanine (GO) repair system of *B. subtilis* [31,44]. Since Mfd is also required to generate adaptive Leu^+^ revertants [22], we investigated whether Mfd works in coordination with MutY to generate Leu^+^ revertants, or if these proteins contribute through independent mechanisms to these mutagenic events.

**Figure 3:**
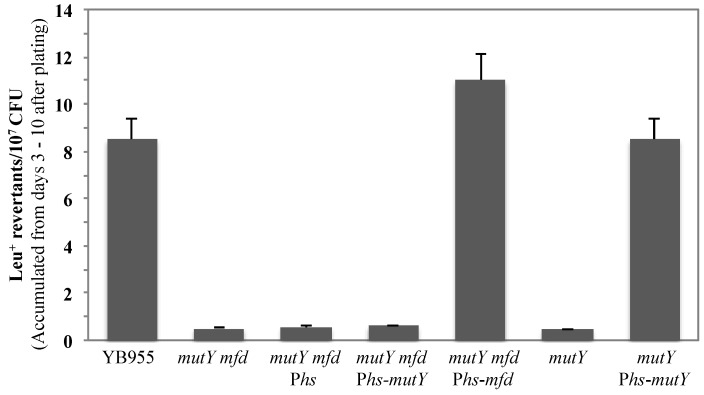
MutY and Mfd act in the same mutagenic pathway. Stationary-phase Leu^+^ reversions in the YB955 (parental strain), PERM818 (*mfd mutY*), PERM1046 (*mfd mutY amyE::*P*hs*), PERM995 (*mfd mutY amyE::*P*hs-mutY*) PERM1042 (*mfd mutY amyE::*P*hs-mfd*), PERM704 (*mutY*) and PERM899 (*mutY* *amyE*::P*hs-mutY*) *B. subtilis* strains were determined as described in Material and Methods. Data represent the average of three separate tests ± standard error of the mean (SEM).

To this end, we constructed a series of strains deficient in both *mutY*, and *mfd*, as well as an expression system for either *mfd* or *mutY* under the transcriptional control of an IPTG-inducible promoter. As shown in Figure 3, with respect to the YB955 (parental strain), the *mutY mfd* strain completely abrogated the production of Leu^+^ revertants in starved *B. subtilis* cells. Notably, expression of Mfd from the IPTG-inducible P*spac* promoter significantly increased the reversion levels of the *leuC* gene. In marked contrast, expression of MutY in the same background did not result in increased Leu^+^ reversion levels (Figure 3). The IPTG-inducible *mutY* construct used in these experiments restored the Leu^+^ mutagenesis in *mutY* cells (Figure 3 and [31]), therefore, we can rule out possible defects in the inducible MutY construct. The lack of response when MutY was overexpressed suggested that production of *leuC* reversion through MutY is dependent on a functional Mfd. In addition, the response observed when Mfd was overexpressed supports the idea that Mfd employs alternative pathways to promote stationary-phase mutations independently of MutY.

Two-hybrid assays were conducted to determine whether MutY directly interacted with Mfd to produce Leu^+^ mutations. In this assay system, PERM1072 was constructed as a target (pTRG-*mfd*) and PERM1084 as bait (pBT-*mutY*) (Table 1). The positive control demonstrated an interaction, which manifested by abundant growth on screening medium (SM) after transforming the reporter cells with the pTRG-Gal11P and the pBT-LGF2 plasmids. To rule out self-activation induced by the transformation of recombinant pBT or pTRG in the BacterioMatch reporter strain, transformations were performed using either recombinant pBT-*mutY*, and the empty pTRG or pBT vector and recombinant pTRG-*mfd*. No significant growth of colonies on screening plates was observed in either case (Figure S2). All positive and negative controls provided expected results. Interestingly, when the interaction pair was switched (pTRG-*mfd* and pBT-mutY), no growth of the *E. coli* indicator strain was detected on the screening medium (Figure S2) suggesting a lack of direct interaction between MutY and Mfd proteins. Of note, the absence of growth on screening medium cannot be attributed to deficient reporter strain transformation. In all cases, abundant growth of colonies was observed on non-screening medium (NSM) (Figure S2).

### 3.3. MutYand UvrA Act in Independent Stationary-Phase Mutagenic Pathways that Require Mfd

**Figure 4:**
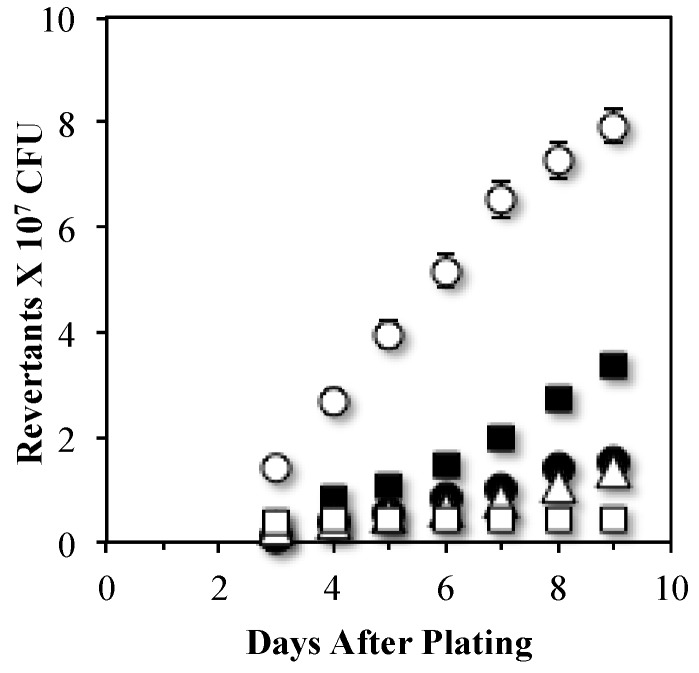
MutY- and NER-dependent SPM pathways operate through Mfd. Frequencies of stationary-phase reversions for *leu* mutant allele of the *B. subtilis* strains YB955 (parental strain) (○), PERM687 (*uvrA*) (■), PERM704 (*mutY*) (●), PERM1352 (*uvrA mutY*) (Δ) and PERM1353 (*uvrA, mutY mfd*) (□) were obtained as described in Materials and Methods. Data represent the average of three separate tests ± standard error of the mean (SEM).

Because Mfd was central to mechanisms that result in Leu^+^ mutagenesis through UvrA and MutY, we tested whether these proteins are in the same mutagenic route. To this end, we estimated mutation rates in backgrounds lacking *uvrA* and/or *mutY*. The results showed that deletion of *mutY* significantly reduced the levels of Leu^+^ revertants produced by the *uvrA* mutant; in fact as shown in Figure 4, the reversion values obtained in the UvrA MutY-deficient strain were similar to those presented by the single *mutY* mutant. Interestingly, disruption of Mfd in the *uvrA mutY* genetic background almost completely abolished the production of Leu^+^ revertants (Figure 4). Altogether, these results provide genetic evidence that UvrA and MutY act in independent mutagenic pathways that requires the participation of Mfd.

### 3.4. Mfd is Highly Expressed during Stationary Phase of B. subtilis

**Figure 5:**
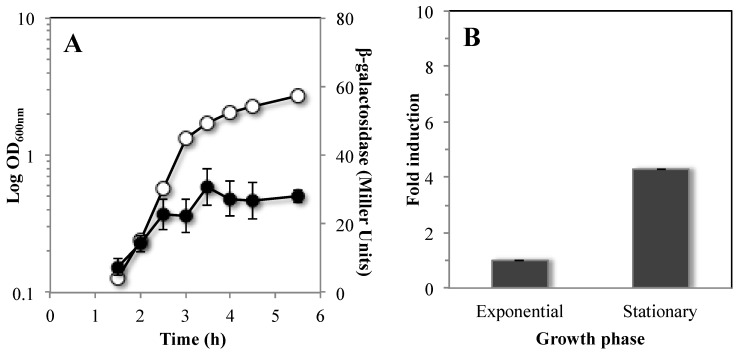
Analysis of Mfd expression during the growth cycle of *B. subtilis.* (**A**) Levels of β-galactosidase of a *mfd-lacZ* transcriptional fusion were measured during the exponential and stationary phase of growth. *B. subtilis* strain PERM1123 was grown in liquid antibiotic (A3) medium. Cell samples were collected at the indicated times and treated with lysozyme, and the extracts were assayed for β-galactosidase as described in Materials and Methods. Data shown are average values from triplicate independent experiments SD for β-galactosidase specific activity (●) and for A_600nm_ values (○). (**B**) Relative fold change of *mfd* mRNA levels in YB955 strain determined by qRT-PCR assays. Levels of mRNA from cultures in exponential growth were compared to stationary phase mRNA levels. The *veg* gene was used to standardize expression. The data shown are average values for triplicate independent experiments ± standard deviations (SD).

The mutagenic events mediated by Mfd have mainly been associated to the stationary phase of growth [22,45]; therefore, we first analyzed the temporal pattern of expression of a transcriptional *mfd-lacZ* fusion inserted into the *mfd* locus of strain YB955. To this end, the strain PERM1123 (*mfd*-*lacZ*) was propagated in Penassay broth (PAB) medium and assayed for *mfd*-driven expression of β-galactosidase during both exponential and stationary phases of growth. Expression of the reporter gene took place in all stages of growth; however, such levels were significantly higher during the stationary phase of growth (Figure 5A). Expression of *mfd* was further analyzed by real-time polymerase chain reaction (qPCR) assays. The results showed that the amount of *mfd* mRNA detected in stationary phase (T_90,_ 90 min after the time point in the culture when the slopes of the logarithmic and stationary phases of growth intercepted) was four times higher than that observed in exponentially growing cells (Figure 5B).

## 4. Discussion

Mfd has been extensively studied in actively growing cells in the context of high-fidelity repair and as a factor influencing replisome progress through obstacles [21] and origin-independent replisome assembly [7]. The results presented here provide insights into yet other Mfd-dependent cellular functions; those that generate mutations in non-replicating and nutritionally stressed cells (SPM). First, our observations suggest that Mfd-dependent mutagenic functions proceed partly through its interactions with proteins of the NER system, since inactivation of *uvrA* partly decreases mutagenesis. A second set of results has also revealed that Mfd is flexible to indirectly coordinate base excision repair events that culminate in SPM.

Based on the results where Mfd was epistatic onto UvrA to produce stationary-phase Leu^+^ revertants (Figure 1), we postulate that an error-prone mechanism involving Mfd and components of the NER system can be activated by DNA lesions (e.g., pyrimidine dimers or abasic sites) in the transcribed strand that stall the transcriptional machinery. In this mechanism, Mfd would be necessary to disassemble the stalled RNA polymerase and to recruit the UvrABC system that excises and eliminates the lesions. In support of this mechanism, a recent report showed that Mfd promotes NER-dependent mutagenic events in the lagging-DNA strand in actively growing *B. subtilis* cells [21]. Within this pathway it is also possible that a transcribing RNAP may pause at a protein–DNA complex that would recruit the NER system to initiate a gratuitous repair reaction that introduces a mutation. Indeed, a previous report in *E. coli* have revealed that even undamaged DNA can be used as a substrate by the NER machinery [46].

Our results also showed that Leu^+^ mutagenesis promoted by Mfd required the activity of PolI (Figure 2). Consistent with the notion that PolI may carry out error-prone DNA synthesis, a previous report showed that, unlike in *E. coli*, *B. subtilis* PolI lacks proofreading activity [33,47]. Moreover, previous reports have also indicated that PolI promote mutagenic events even in the absence of exogenous DNA damage [33,48].

The contribution of the NER pathway to Mfd-mediated mutagenesis was partial. The absence of UvrA decreased SPM but not to the levels observed in the single *mfd* mutant (Figure 1). This pointed to the possibility that Mfd could interact with other factors in the formation of stationary-phase mutations. A recent study showed that processing of A-G/C mismatches that accumulate in starved *B. subtilis* by the DNA glycosylase MutY is involved in generating SPM-associated Leu^+^ reversions [31]. Results obtained in this work strongly suggest that the MutY-dependent mutagenic event leading to generation of Leu^+^ revertants requires the participation of Mfd (Figure 3). Even more interesting were the results from the epistatic analysis performed with Mfd, UvrA and MutY (Figure 4) suggesting the participation of these proteins in independent pathways that require Mfd to promote mutagenic events in nutritionally stressed *B. subtilis* cells.

On the basis of current and previous results, we propose a model for production of reversions in the *leuC427* allele resulting in a Leu^+^ prototrophic phenotype in non-growing/non-replicating *B. subtilis* cells. A-G/C mismatches (recognized by MutY) generated in non-growing cells [31] are processed by MutY to eliminate adenine from the mismatch and generate an AP site. However, due to the high cytotoxicity of AP sites [18], it has been shown that MutY remains tightly bound to this lesion to protect the DNA from further damage until yet-to-be determined downstream components promote the release of MutY to allow repair of the AP site by BER components [49,50]. Thus, we speculate that the MutY-AP site complex stalls RNA polymerase during transcription of *leuC427* and activates the Mfd-dependent event to generate the A to G reversion in this allele [26]. In this context, the function of Mfd is then required to dissociate the transcription elongation complex through its interaction with the RNA polymerase followed by recruitment of excision and DNA repair synthesis activities. This model is in agreement with the results that ruled out a direct interaction between Mfd and MutY (Figure S2) and explains why the production of Leu^+^ revertants by MutY requires the participation of Mfd (Figure 3). Since NER is partially involved in *leuC* mutagenesis (Figure 1), a BER-dependent mechanism mediated by the AP endonucleases Nfo, ExoA and/or Nth and error-prone synthesis by PolI could also complete the error-prone repair event. In support of this notion, previous reports have shown that the mismatch repair machinery becomes suppressed during stationary phase which would increase A-G/C mismatches [38]. It was also recently found that AP sites that accumulate in stationary-phase cells are processed by Nfo, ExoA and/or Nth in an error-prone manner by the DNA polymerase PolX [19].

No significant differences in the production of growth-associated Leu^+^ revertants were observed between the parental strain YB955 and mutants of the Mfd-UvrA-PolA-dependent pathway described in this work (Table S1). These results support the concept that the Mfd-NER mediated mutagenic events are specific to conditions in which DNA replication and cell growth are limiting. A trademark of stationary-phase *B. subtilis* cells is the development of differentiated sub-populations (e.g., the cells that develop competence are different from those that give rise to endospores). These subpopulations form under conditions in which DNA replication and cell division are halted [23,24,25] and require the activation of specific genetic programs (K and Spo transcription factors). A recent report on the role of Mfd in the population of cells that develop into spores demonstrated that this factor was important for efficient production of spores but prevented the formation of mutations [34]. The latter is the opposite of what occurs in the rest of stationary-phase cells. These observations are significant because they provide support for the concept that a stressed *B. subtilis* culture differentiates cells to either increase genetic diversity or to protect genome integrity and develop dormancy. In regards to Mfd, these observations substantiate the idea that this factor may be both a repair and transcription modulation factor, and therefore may have different roles in vivo.

In a much broader perspective, transcription-mediated mutagenesis allows the study of genetic processes in cells under mitotic arrest. Further, the factors governing transcription-associated mutagenesis have been associated with antibiotic resistance in bacterial pathogens, the development of neoplasia as well as other human diseases [51,52]. In addition, these factors have been linked to the formation of transient phenotypes and the development of certain cancers [52,53,54]. In bacteria, the factors studied here have recently been implicated in the acquisition of fluoroquinolone-resistant mutations or the ability to withstand higher inhibitory doses of several antibiotics [51,55]. Therefore, further study of these factors has a great potential for developing therapeutic targets to mitigate the increase in antibiotic resistance, as well as treat developmental diseases in humans. From an evolutionary standpoint, mechanisms such as transcription-mediated mutagenesis may help to explain how a population of pathogenic cells produces genetic diversity during chronic infections [56].

## 5. Conclusions

We propose that Mfd promotes mutagenesis in starved *B. subtilis* cells by coordinating both known (NER) and previously unknown (MutY and PolI) associated repair pathways and support the concept that stressed cells bias the production of genetic diversity towards highly transcribed regions in the genome.

## Figures and Tables

**Table 1:** *B. subtilis* strains and plasmids used in this study.

**Strain**	**Relevant genotype**	**Reference or source**
YB955	*hisC952 metB5 leuC427 xin-1 SpSENS*	[26]
YB9801	YB955 *mfd*::*tet*, Tet ^r^	[22]
YB9900	YB955 *uvrA::em*, Em ^r^	^a^ BSU35160 → YB955
JJS164	TF8a Δ*polA::sp*, Sp ^r^	[33]
AMP100	YB955 Δ*polA::sp*, Sp ^r^	^a^ JJS164 → YB955
AMP101	YB955 Δ*polA::sp*, *mfd::tet*, Sp ^r^ Tet ^r^	^a^ JJS164 → YB9801
BSU35160	168 *uvrA::em*, Em ^r^	*Bacillus* Genetic Stock Center (BKE Knock-out collection)
HAM300	YB955 *uvrA::emmfd*::*tet*, Em ^r^ Tet ^r^	^a^ YB9900 → YB9801
PERM598	168 *mutY*::*sp*, Sp ^r^	[31]
PERM687	YB955 *uvrA*::*sp*, Sp ^r^	Peter Setlow
PERM704	YB955 *mutY*::*sp*, Sp ^r^	[31]
PERM899	YB955 *mutY*:: *sp* *amyE*::P*hs-mutY*, Cm ^r^	[31]
PERM818	YB955 *mfd*::*tet mutY::sp*, Tet ^r^ Sp ^r^	^a^ PERM598 → YB9801
PERM995	YB955 *mfd*::*tet mutY*::*sp amyE*::P*hs-mutY*, Cm ^r^	^b^ pPERM852 → PERM818
PERM1029	YB955 *mutY::em*, Em ^r^	^b^ pPERM979 → YB955
PERM1041	YB955 *mfd*::*tet mutY::em*, Tet ^r^ Em ^r^	^b^ pPERM979 → YB9801
PERM1042	YB955 *mfd*::*tet mutY*::*em amyE*::*Phs-mfd*, Tet ^r^ Em ^r^ Sp^r^	^b^ pPERM1041 → PERM1041
PERM1046	YB955 *mfd*::*tet mutY*::*em amyE*::*Phs*, Tet ^r^ Em ^r^ Sp ^r^	^b^ pdrE *amyE* → PERM1041
PERM1352	YB955 *mutY*::*em uvrA*::*sp*, Em ^r^ Sp ^r^	^a^ PERM1029 → PERM687
PERM1353	YB955 *mfd*::*tet mutY*::*em uvrA*::*150*, Tet ^r^ Em ^r^	^a^ PERM1029 → PERM1352
PERM1123	168 pMUTIN4::*mfd-lacZ*, Em ^r^.	[34]
**Plasmids**	**Description**	**Reference or source**
pHyperspank	Integrative vector, Sp ^r^	David Rudner
pDR244	*cre*, temperature-sensitive replication Amp ^r^ Sp ^r^	Bacillus Genetic Stock Center (BKE Knock-out collection)
pPERM852	pHyperspank-*mutY* Integrative vector with *mutY* control by IPTG, Amp ^r^ Cm ^r^	[31]
pPERM979	pMUTIN4::*mutY*, Amp ^r^ Em ^r^	This study; [35]
pPERM1043	pHyperspank-*mfd* Integrative vector with *mfd* under the control of IPTG, Amp ^r^ Sp ^r^	This study
pPERM1084	pBT-*mutY*, Cm ^r^	This study
pPERM1072	pTRG-*mfd*, Tet ^r^	This study

^a^ Chromosomal DNA from the strain to left of the arrow was used to transform the strain to the right of the arrow; ^b^ DNA of the plasmid to the left of the arrow was used to transform the strain to the right of the arrow; Amp, ampicillin; Cm, chloramphenicol; Em, erythromycin; Neo, neomycin; Sp, spectinomycin; Tet, tetracycline; ^r^ Denotes phenotype of antibiotic resistance.

## Supplementary Materials

The following are available online at www.mdpi.com/2073-4425/7/7/33/s1, Figure S1: survival of strains used in stationary phase assays subjected to leucine starvation, Figure S2: Bacterial two-hybrid screening to detect physical interactions between MutY and Mfd, Table S1: The spontaneous Leu^+^ reversion rates for YB955 and derivatives.

## Abbreviations

The following abbreviations are used in this manuscript:

A, G, C	adenine, guanine and cytosine	3-AT	3-amino-1,2,4-triazole
Amp	ampicillin	AP	apurinic/apyrimidinic
BER	base excision repair	CFU	colony-forming unit
Cm	chloramphenicol	Ct	threshold cycle
DNA	deoxyribonucleic acid	DO	optical density
Em	erythromycin	GO	oxidized guanine
HCl	hydrochloric acid	IPTG	isopropyl-β-Dthiogalactopyranoside
LB	Luria-Bertani	Leu^+^	leucine reversions
MMR	Mismatch repair	NER	nucleotide excision repair
NSM	non-screening medium	ONPG	ortho-notrophenyl-β-D-galactopyranoside
ORF	open reading frame	PAB	Penassay broth
PCR	Polymerase chain reaction	RNA	Ribonucleic acid
RNAP	ribonucleic acid polymerase	SEM	standard error of the mean
SM	screening medium	SMM	spizizen minimal medium
SMS	Spizizen minimal salts	Sp	spectinomycin
SPM	stationary-phase mutagenesis	T_90_	90 min after the cessation of exponential growth
TBAB	tryptose blood agar base	TCR	transcription-couple repair
Tet	tetracycline	UV	ultraviolet light
