# Supplementary material for: Stationary-Phase Mutagenesis in Stressed Bacillus subtilis Cells Operates by Mfd-Dependent Mutagenic Pathways
Source: Genes (Basel). 2016 Jul 5;7(7):33. doi: 10.3390/genes7070033 (PMC4962003; doi:10.3390/genes7070033)
Supplement: Supplementary file 1 [file genes-07-00033-s001.docx]

Supplementary Materials

Stationary-Phase Mutagenesis in Stressed *Bacillus subtilis* Cells Operates by Mfd-Dependent Mutagenic Pathways

Martha Gómez-Marroquín, Holly A. Martin, Amber Pepper, Mary E. Girard, Amanda A. Kidman, Carmen Vallin, Ronald E. Yasbin, Mario Pedraza-Reyes and Eduardo A. Robleto

Materials and Methods

*Survival Rates Measurements*

The survival rates of the bacteria plated on the minimal selective medium were determined as follows [24]. Three agar plugs were removed daily from selective plates using in stationary phase assays. The plugs were removed with sterile Pasteur pipettes and taken from areas of the plates where no growth of revertant colonies was observed. The plugs were suspended in 1 mL of 1 × Spizizen salts, mixed, diluted, and plated on SMM containing all the essential amino acids (50 µg mL^−1^). The number of colonies was determined following 48 h of growth at 37 °C.

*Bacterial Two-Hybrid Analysis of Protein-Protein Interactions*

The *E. coli* BacterioMatch Two-Hybrid System (Stratagene, La Jolla, CA, USA) was used to investigate in vivo interactions between MutY and Mfd proteins according to the instructions provided by the manufacturer [1–3]. This system is based on the transcriptional activation, where a protein of interest (target, MutY) is fused to the bacteriophage λcI protein which binds to the λ operator sequence. The other protein of interest (bait, Mfd) is fused to the N-terminal domain of RNA polymerase α subunit (Table 1). If MutY and Mfd proteins interact with each other, RNA polymerase recruited to the promoter causes transcriptional activation of the HIS3 reporter gene. The resulting activation is detected by the formation of colonies on agar medium (SM) plates lacking histidine and containing 5 mM of 3-amino-1,2,4-triazole (3-AT), 12.5 μg mL^−1^ tetracycline, 25 μg mL^−1^ chloramphenicol and 12.5 μg mL^−1^ IPTG. Transformation efﬁciency is determined by the number of colonies that grown on non-screening medium (NSM) lacking 3-AT.

According to the kit instructions, BacterioMatch II Validation Reporter Competent Cells were employed as the reporter strain. Transformation of reporter strain with pBT-LGF2 and pTRGGal11P were used as a positive interaction control, these cells scored positive for growth on the selective screening medium. Cells transformed with a recombinant plasmid PERM1084 or PERM1072 (pBT-*mutY* and pTRG-*mfd* respectively) and an empty vector (pBT or pTRG) were used as negative control. Interaction assays between Mfd and MutY were performed by transforming reporter cells with PERM1084 and PERM1072 plasmids (pBT-*mutY* and pTRG-*mfd*) (Table 1).


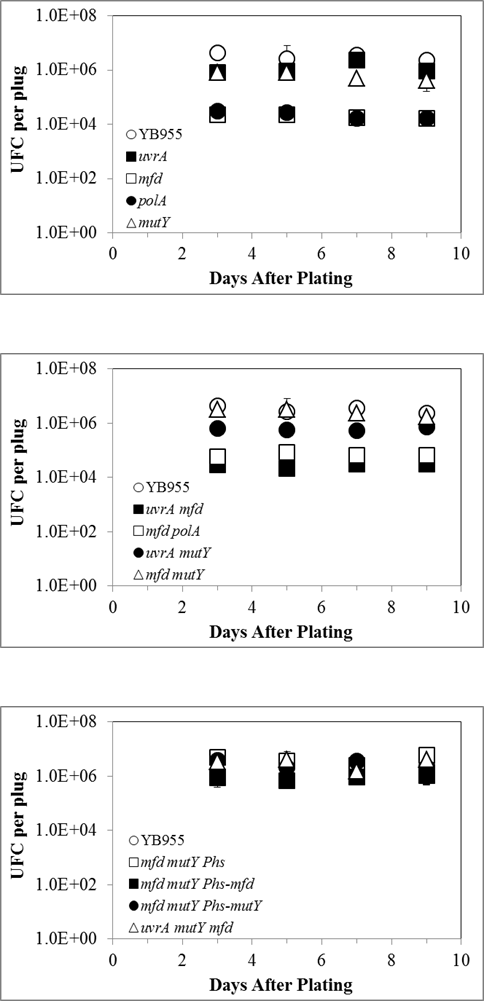


**Figure S1**. Survival of strains used in stationary phase assays subjected to leucine starvation. Three plugs of bacteria containing agar were taken from zones of the selection plates where no growth of revertant colonies was observed. Titers were determined on media containing all essential amino acids every other day for testing of viability of non-revertant background cells. Results presented are average values ± SD from three independent experiments.


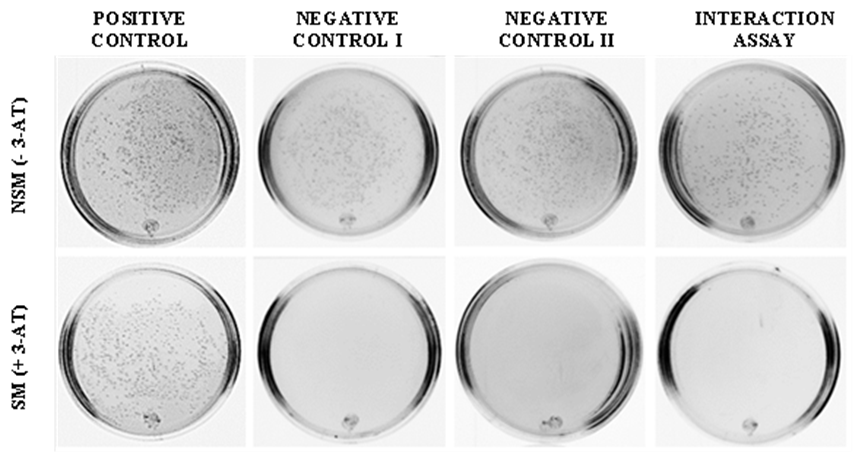


**Figure S2**. Bacterial two-hybrid screening to detect physical interactions between MutY and Mfd. Top panel, transformants plated on Non-selected Screening medium (NSM) lacking 3-amino-1,2,4-triazole (3-AT). Bottom panel, transformants selected on Screening Medium (SM) plates containing 5 mM 3-AT. Positive Control, transformants containing pBT-LGF2 and pTRG-Gall lp plasmids. Negative Control I, transformants containing pBT-*mutY* (PERM1084) and pTRG plasmids and Negative Control II, transformants containing pBT and pTRG-*mfd* (PERM1072) plasmids*.* Interaction assay, transformants containing pBT-*mutY* (PERM1084) and pTRG-*mfd* (PERM1072) plasmids. See Material and Methods section for more details.

**Table S1**. The spontaneous Leu^+^ reversion rates for YB955 and derivatives.

| Strain | Mutation Rate | *Confidence Value |
| --- | --- | --- |
| YB955 | 2.35 E-08 | ±0.81 |
| *mfd* (YB9801) | 1.92 E-08 | ±1.23 |
| *polI* (AMP100) | 2.14 E-08 | ±1.33 |
| *uvrA* (PERM687) | 2.00 E-08 | ±0.12 |
| *mutY* (PERM704) | 0.34 E-08 | ±0.13 |
| *uvrA mutY* (PERM1352) | 0.19 E-08 | ±0.10 |
| *mfd uvrA* (HAM300) | 1.70 E-08 | ±1.12 |
| *mfd mutY* (PERM1041) | 0.18 E-08 | ±0.25 |
| *mfd mutY* *amyE*::P*hs* (PERM1046) | 0.15 E-08 | ±0.42 |
| *mfd mutY* *amyE*::P*hs*-*mutY* (PERM995) | 0.27 E-08 | ±0.33 |
| *mfd mutY* *amyE*::P*hs*-*mfd* (PERM1042) | 0.25 E-08 | ±0.04 |

*Error is representative of the 95% confidence value.

**References**

1. Khoo, S.K.; Loll, B.; Chan, W.T.; Shoeman, R.L.; Ngoo, L.; Yeo, C.C.; Meinhart, A. Molecular and structural characterization of the PezAT chromosomal toxin-antitoxin system of the human pathogen *Streptococcus pneumoniae*. *J. Biol. Chem.* 2007, *282*, 19606–19618.
2. Mishra, M.N.; Kumar, S.; Gupta, N.; Kaur, S.; Gupta, A.; Tripathi, A.K. An extracytoplasmic function sigma factor cotranscribed with its cognate anti-sigma factor confers tolerance to NaCl, ethanol and methylene blue in *Azospirillum brasilense* Sp7. *Microbiology* 2011, *157*, 988–999.
3. Tschowri, N.; Lindenberg, S.; Hengge, R. Molecular function and potential evolution of the biofilm-modulating blue light-signalling pathway of *Escherichia coli*. *Mol. Microbiol.* 2012, *85*, 893–906.
